# An Implementation Log for quantitatively tracking key implementation variables in near real time: Development, qualitative evaluation, and demonstration in a clinical trial conducted in community mental health

**DOI:** 10.21203/rs.3.rs-6107938/v1

**Published:** 2025-09-10

**Authors:** Laurel Sarfan, Linyan Ge, Emma R. Agnew, Marlen Diaz, Krista Fisher, Shayna A. Howlett, Rafael Esteva Hache, Julia Spencer, Allison G. Harvey

**Affiliations:** Kaiser Permanente Southern California; University of California Berkeley; University of California Berkeley; University of California Berkeley; University of California Berkeley; University of California Berkeley; University of California Berkeley; University of California Berkeley; University of California Berkeley

**Keywords:** implementation, implementation log, tracking, planning, facilitation, participatory, community mental health

## Abstract

**Background:**

Implementation is complex and not always reported in detail, presenting barriers to replication and translatability. The goals herein were to: (1) describe the development of an Implementation Log that yields quantitative data on a wide range of key implementation variables through near real time tracking; (2) gather qualitative feedback from study personnel, and (3) demonstrate ways the log data could be analyzed.

**Method:**

These goals were executed in the context of a parent hybrid effectiveness-implementation cluster randomized controlled trial to evaluate a sleep treatment (Transdiagnostic Intervention for Sleep and Circadian Dysfunction) implemented in community mental health centers via facilitation. The Implementation Log was derived from prior research on implementation tracking; reporting guidelines, frameworks, and taxonomies; piloting; and collaboration with facilitators in the trial. After development, the log was used for 17 months by study personnel (i.e., facilitators; *N*=6). Then, the facilitators were interviewed about their perceptions of the log’s acceptability, appropriateness, and feasibility. Interviews were deductively and inductively coded. To demonstrate possible uses for the log, two sets of preliminary analyses were conducted. First, we quantified the most time-intensive events, targets, implementation strategies, and intended outcomes tracked by facilitators. Second, we quantified the most time-intensive events, implementation strategies, and targets directed toward the most time-intensive outcomes, to unpack relationships *between* implementation variables.

**Results:**

Facilitators tracked almost 4,000 hours of events, targets, and outcomes using the Implementation Log. Facilitators appreciated the log’s value to the field but had three main suggestions: (1) automatize the format, (2) offer more training and supervision, and (3) improve flexibility to adjust the log’s variables. Per quantitative analyses, facilitators spent the most time on the event of internal team meetings for problem solving and sharing updates, the target of executing, and the implementation strategy of facilitation. Additionally, events, targets, and strategies were identified for the most time-intensive outcomes – specifically, adoption, penetration, and fidelity.

**Conclusions:**

This report describes a novel Implementation Log for quantitatively tracking a range of key implementation variables in near real time. Although modifications may improve end-user experience, the log has potential to improve the reporting, planning, and analysis of implementation efforts.

## Introduction

Implementation science seeks to bridge the gap between research and practice. A growing scientific literature has evaluated a broad range of implemented interventions with encouraging outcomes (1,2). However, the processes involved in implementation are often complex and not always described in detail, presenting barriers to replication and translatability (3). Thus, calls have been made to **(1)** improve the rigor and consistency with which implementation is reported (4,5) and **(2)** untangle relationships between implementation variables (e.g., strategies and outcomes) in the context of ‘real world’ implementation studies (6).

In response, we developed an Implementation Log for tracking key implementation variables. The Implementation Log and its development are described in detail in the Method section. In brief, building on prior research, the log was developed following Proctor et al.’s (4) guidelines to report the following variables when conducting research related to implementation strategies: action, actor, action target, temporality, dose, implementation outcomes, and justification. In addition to grounding the Implementation Log in these recommendations, each variable tracked by the log was derived through piloting, collaboration with facilitators, and/or standardized frameworks, taxonomies, and guidelines from implementation science (see Method).

The log development was conducted in the context of a ‘parent’ cluster randomized, hybrid type 2 effectiveness-implementation trial within community mental health centers (CMHCs). In the parent trial, the overarching implementation approach was external facilitation. A team of facilitators worked with the CMHCs to help implement two versions of the treatment of interest: the Transdiagnostic Intervention for Sleep and Circadian Dysfunction (TranS-C) for people with serious mental illness (see protocol (7) and Method for additional details about facilitators). Facilitation was selected as the overarching approach based on promising evidence that it can support organizations with implementing new interventions (8,9). To date, a handful of studies have characterized the specific activities involved in facilitation (10,11) and tested elegant approaches to track fidelity to facilitation (12) and evaluate the time and cost spent on facilitation activities (13,14). However, to our knowledge, facilitation tracking tools that log a wider range of key implementation variables – for instance, implementation targets, strategies, and outcomes – have not yet been developed.

Indeed, detailed yet practical tools to track implementation are critical to addressing challenges in implementation science. In particular, such tools enable rigorous tracking of complex implementation projects (4,5). In turn, the resulting data can be used to analyze relationships between a range of key implementation variables (6). In the implementation literature, innovative studies have piloted approaches to plan for, track, and assess implementation.

In one subset of the literature on implementation tracking, methods have been developed that leverage qualitative data. For example, Bunger et al. (15) developed ‘activity logs’ completed by implementation personnel once a month. These logs were qualitatively coded by the research team to determine which implementation strategies had been used. As another example, Boyd et al. (16) qualitatively coded audio recordings of implementation team meetings to identify use of key implementation variables. In a related study, implementation team discussions or ‘periodic reflections’ were conducted regularly regarding implementation processes (e.g., progress, context, adaptations, barriers), and which were then qualitatively coded for key implementation variables (17). Other studies focused on assessing implementation processes have qualitatively coded archival materials, such as annual reports and study proposals (18,19). Such qualitative methods are critical for an in-depth understanding of complex implementation processes. However, a drawback is that they may be prohibitively time- and resource-intensive – for example, when analyzing large amounts of data or conducting a range of different analyses.

A second important subliterature of implementation tracking has used methods that focus on delving deeply into a dimension of implementation. For example, the Framework for Reporting Adaptations and Modifications to Evidence-based Implementation Strategies (FRAME-IS) offers a rigorous approach to document modifications to implementation strategies through a series of modular and detailed checklists (20) (see (21) and (22) for other examples of tracking adaptations and modifications to implementation strategies). Related, systematic processes have been developed to carefully assess, plan for, and offer feedback on adaptations to treatments during implementation efforts, such as the Dynamic Adaptation Process (23). Other studies have developed surveys that specifically focus on use of implementation strategies (24), as well as satisfaction with the strategies used (25).

In a third subliterature, researchers have used structured approaches to track a range of implementation variables, generally supplemented with qualitative coding. Walsh-Bailey et al. (26) tested three implementation tracking tools for logging a range of variables. All three approaches used open-ended prompts that required qualitative coding; however, one of the approaches also used a structured, pre-populated format for some variables. This structured, pre-populated format was identified as a strength in semi-structured interviews with end users. Another valuable approach, the Pragmatic Implementation Reporting Tool (3), helps implementation teams specify the implementation strategies used in a given project, then characterize the strategies in greater detail per the Proctor et al. (4) reporting recommendations (e.g., actor, action, dose, target, outcomes). This tool also offers a blend of structured, pre-populated response options paired with open-ended response options.

Each of these existing approaches has unique advantages. However, a gap is the availability of a structured, ‘ready-made’ tracking tool for gathering quantitative data on a wide range of implementation variables to facilitate routine collection and analysis of large quantities of data. Thus, we sought to offer a ready-made tool that would offer the following complementary contributions: **(1)** data that could be analyzed quantitatively and without requiring qualitative coding, **(2)** a log to track a range of key implementation variables (e.g., activities, time, targets, strategies, outcome (4)), while **(3)** tracking in near ‘real time’ (i.e., submitted on a daily or weekly basis).

Further, we aimed to assess perspectives of study personnel using the logs for quality improvement. Then, we sought to demonstrate how using the tool could provide information about key implementation variables and their relationships. Such data have a dual purpose of knowledge-sharing across teams to help with implementation planning (e.g., how and where to allocate resources) *and* helping to systematically report, assess, and unpack the complexities of implementation.

Together, the report herein has three goals: **(1)** describe our development of the Implementation Log, **(2)** describe study personnel’s (i.e., facilitators) perceptions of using the log, and **(3)** demonstrate ways the log data could be used by: (a) reporting time spent on each facilitation event, target, implementation strategy, and outcome, and (b) quantifying the most time-intensive events, implementation strategies, and targets directed toward the most time-intensive implementation outcomes (i.e., to help unpack relationships *between* implementation variables).

## Methods

### Parent Trial Design

The Implementation Log presented herein was developed in the context of a parent cluster randomized hybrid type 2 effectiveness-implementation trial. In brief, CMHCs in 10 counties across California, U.S. were cluster randomized to Standard TranS-C (*n* = 5 CMHCs) or Adapted TranS-C (*n* = 5 CMHCs). More details about the CMHCs can be found in the Supplement. The parent trial consisted of three phases: the implementation phase, the train-the-trainer phase, and the sustainment phase (7,27,28). Because the log was designed to track *implementation*, the present study is limited to the implementation phase. The parent trial was preregistered on clinicaltrials.gov (identifier: NCT04154631) and received approval from the Committee for the Protection of Human Subjects at the University of California, Berkeley. This report followed the Standards for QUality Improvement Reporting Excellence (SQUIRE) 2.0 guidelines (checklist attached as Additional File) (29). The trial was funded by the National Institute of Mental Health (R01MH120147).

### Facilitators

In the parent trial, Standard and Adapted TranS-C were implemented in CMHCs via the overarching approach of external facilitation (30). Specifically, each CMHC received direct support from a lead facilitator (ERA) as well as a team of facilitators (MD, KF, REH, JS, SAH). The lead facilitator was a licensed clinical social worker with experience delivering and supervising clinical services in community mental health. The other facilitators on the team had bachelor’s degrees in psychology or related fields as well as a background or interest in mental health, sleep treatment, and community engagement. They were trained and directly supervised by the lead facilitator and the parent trial’s principal investigator (PI; AGH). All facilitators were employed by the research team.

Initially, the facilitation team consisted of four facilitators (*n* = 1 lead facilitator, *n* = 3 team members). Two facilitators left the study during the implementation phase to pursue advanced degrees and were replaced by *n* = 2 new facilitators. The new facilitators were trained by their predecessors, the lead facilitator, and the PI. The present report includes all six facilitators.

### Implementation Log Development

#### Background.

Initially, facilitators completed a prior iteration of the Implementation Log, used for approximately 10 months. However, completion rates were very low, and facilitators expressed difficulty with completing the log. Aligning with models of participatory research (31), rather than proceeding with this prior version, we used this prior log as a pilot and designed a new version, which incorporated facilitator feedback and was iteratively refined in collaboration with the facilitators. This final version of the Implementation Log is described below. Because of this shift, the first 10 months of the prior log were not included in the quantitative analyses.

#### Final Version.

The final version of the Implementation Log was formatted in Excel (blank template included as Additional File). All variables tracked in the log are described below. As noted above, the log was developed using Proctor et al.’s (4) reporting guidelines. Specifically, facilitators used to the log to track the following variables, as recommended by Proctor and colleagues (4): action, actor, action target, temporality, dose, implementation strategy, and intended outcome. The one exception to following the guidelines is that we did not include a specific variable for tracking justification. Instead, to reduce burden, facilitators were instructed to select implementation strategies based on other variables selected (e.g., event, target, and intended outcome).

Using the Excel file, facilitators completed a new log each day. Their daily log included the date (i.e., for temporality) and the “events,” or actions they completed that day. Each event was logged in a new row. For each event, facilitators entered 11 variables, presented in sequential columns as follows: County; Time; Communication Type; To Whom the Target Applies (shortened to ‘At Whom? (Target)’ in the log); Target; Implementation Strategy 1; Implementation Strategy 2; Implementation Strategy 3; Implementation Strategy 4; Intended Outcome; and To Whom the Outcome Applies (shortened to ‘At Whom? (Outcome)’ in the log). For all variables, standardized options could be selected from dropdown menus. Each variable is described in turn below. Lists of all variable options and most corresponding definitions were included in the log for facilitators to reference (4,32).

#### Event.

When logging the events that they completed each day, facilitators could choose between prespecified event options that fell into five categories: Communication with Providers, Communication with Leadership, Internal Team Meetings/Activities, Supervision/Consultation/Communication with Clients, and Communication with Whole Network. These prespecified event options were intended to capture the primary activities completed by facilitators. They were devised through review of activities that facilitators had reported in the prior version of the log as well as discussions and iterative revisions between the research team and facilitators. The final version of the log initially had 22 pre-specified event options. However, additional events were added as the facilitator roles evolved, including one event specific to the lead facilitator. Thus, by the end of the study, facilitators could choose between 24 prespecified options (25 for the lead facilitator). Three notes here. First, we decided to use prespecified events rather than open entry, because this aligned with primary purpose of the log. Additionally, the prior version of the log had used open entry, and facilitators reported that the cognitive effort and time needed to articulate each event was a barrier to completing the log. Second, the total number of events for analysis was 27 (i.e., two more than the standardized options presented to facilitators). Specifically, to minimize facilitator burden, two additional variables of ‘supervision’ and ‘trainings’ were imported from other trackers maintained by the facilitators. See the Data Cleaning section below for details. Third, as the parent study evolved, changes were made to the log. For instance, when the study shifted to focus on the train-the-trainer phase, events were added that focused on train-the-trainer. However, because the focus of the present report was on implementation, events specifically related to train-the-trainer were excluded from the final analyses herein.

#### County.

Facilitators selected the county or counties on which a given event was focused.

#### Time.

Facilitators entered the time spent on each event. They could manually enter time as a numerical value, or they could select from a drop-down menu of 30-minute increments ranging from “<30 min” to “>5 hours.”

#### Communication Type.

Facilitators selected the communication format of the event. The final options for this variable were: phone, email, face-to-face, Zoom, or other[1]. An additional option of ‘in person’ was added for analysis when variables were merged in from the other trackers (see below).

#### Target.

Target assessed *at what* the event was directed (e.g., barrier, facilitator). As suggested by Proctor et al. (4), the list of targets from which facilitators could select consisted of the lower-order constructs from the Consolidated Framework for Implementation Framework (CFIR) (33). For instance, a facilitator may have conducted a *training (event)* to increase *knowledge and beliefs about the intervention (target from CFIR)*. See the ‘Lists + Definitions’ tab of the Implementation Log in the Supplement for all CFIR lower-order constructs and definitions.

#### At Whom (Target).

At Whom (Target) assessed *at whom* the target was directed. The options were: patients, providers, leadership, UC Berkeley Team, CMHC as a whole, or other. Additionally, from manual entries by facilitators, a category of ‘champions’ was added during data processing.

#### Implementation Strategy 1, 2, 3, and 4.

Facilitators could select up to four implementation strategies associated with each event. Implementation strategies indicated *how* facilitators were enacting an event to achieve an intended outcome. As with all variables, facilitators could select from a drop-down list of implementation strategies. This list was derived from three sources. As the primary source, all strategies were included from the Expert Recommendations for Implementing Change (ERIC) taxonomy of implementation strategies (34). Then, to capture strategies relevant to facilitation in CMHCs, fifteen additional strategies were identified in partnership with facilitators and drawn from the integrated Promoting Action on Research Implementation in Health Services framework (30) and a study focused on developing a community-academic-clinical partnership grounded in community-based participatory research (35). See ‘Lists + Definitions’ tab of example log in Supplement for all strategies. As an example of implementation strategy tracking, a facilitator may have engaged in *initial outreach to providers (event) using a variety of communication methods (implementation strategy)*. Note that ‘train-the-trainer’ was included in the log as an implementation strategy; however again, given that the focus of this report is the implementation phase, this strategy was not included in the quantitative analyses.

#### Intended Outcome.

Facilitators selected the primary outcome they were aiming to impact with each event, from Proctor’s taxonomy of implementation outcomes (36). This taxonomy identifies and operationalizes eight implementation outcomes: adoption, acceptability, appropriateness, feasibility, fidelity, implementation cost, penetration, and sustainability. As an example, a facilitator may have engaged in *patient troubleshooting (event)* to improve *feasibility (outcome)*.

#### At Whom (Outcome).

At Whom (Outcome) assessed *at whom* the outcome was directed. The options were: patients, providers, leadership, UC Berkeley Team, CMHC as a whole, or other.

#### Templates.

In the Implementation Log Excel spreadsheet, there was a sheet labeled ‘Templates.’ On this sheet, facilitators were provided with examples to demonstrate how to track the above variables. Additionally, there were numbered sheets, which corresponded to each of the prespecified events. On each of these numbered sheets, several likely options were presented for all the variables described above, with the most likely option/s highlighted in yellow. These were included to help facilitators with decision-making, based on facilitator feedback that selecting from the possible options for each variable felt overwhelming and time consuming.

### Qualitative Semi-Structured Interview Collection and Coding

To assess facilitators’ perceptions of the Implementation Log (Aim 1), a semi-structured interview guide was developed by the first, second, and last author. The interview consisted of five questions plus standardized probes to assess perceptions of the log with respect to acceptability, appropriateness, and feasibility (36). See Appendix A for the interview guide.

The interviews were conducted by the first and second author, who also served as the lead coder (LDS) and co-coder (LG), respectively. These roles were assigned based on qualitative research experience. The interviews were conducted after all recruitment and most study visits for the implementation phase had been completed. All interviews were conducted on HIPAA-compliant video platform, recorded, and transcribed (mean interview length (range) = 26 min (18 to 33 min)). Transcriptions were corrected by the lead coder and co-coder.

The interviews were coded in two stages. In the first stage, the interviews were *deductively* coded for facilitators’ perceptions of acceptability, appropriateness, and feasibility (36). Average percent agreement was high (88.7%). In the second stage, *inductive* coding was used to identify themes that emerged from the interviews. The lead coder and co-coder devised an inductive codebook after reading the interviews multiple times. This codebook consisted of overarching *categories* and *themes* within each category. The categories were: positive characteristics/likes, barriers/dislikes, and suggestions for improvement. Across the categories, 34 themes were identified (see Table 1). Next, both coders coded all interviews using the codebook (range of Cohen’s kappa for inductive codes = 0.72 to 0.94) (37).

### Quantitative Data Cleaning

Facilitators used the final version of the Implementation Log from March 1, 2021 to October 13, 2023. For the present study, analyses were limited to entries made from March 1, 2021 to August 31, 2022 (17 months total). This period started with the shift to the new version of the log (March 1, 2021) and ended when the patient enrollment for the implementation phase had finished (August 31, 2022). Originally there were 8,337 rows of events logged by facilitators. After cleaning, 5,892 rows were left in the final version. In the paragraphs below, key details about the cleaning process are described. See Additional File for more details.

For ‘Time,’ the standardized entries from the dropdown menu were <30 min, 30 min to 1 hr, 1 to 1.5 hrs, 1.5 to 2 hrs, 2 to 2.5 hrs, 2.5 to 3 hrs, 3.5 to 4 hrs, 4.5 to 5 hrs, and > 5 hrs. Alternatively, facilitators could manually enter time. When facilitators used standardized entries, we took the midpoint of the range (or the maximum of 5 hrs if selected). Similarly, when facilitators manually entered a range, we took the midpoint (e.g., ‘3 to 3.5hrs’ was converted to ‘3.25’ hours). For all analyses, time was analyzed in the unit of hours.

Note that although 92.0% of the data analyzed for the present study came from the Implementation Log, data were collated from two additional sources: (1) a facilitator training/workshop log and (2) a supervision log. The purpose of collating the logs was to reduce burden on the facilitators by integrating other logs that they were already maintaining. More details on collating process can be found in the Additional File.

### Quantitative Planned Analyses

All analyses were conducted in Python Version 3.9 using interactive development environment JupyterLab. The main packages used were: pandas, matplotlib, numpy, json. datetime, and re.

For Aim 2a, we report the proportion of time spent on each event, target, outcome, and strategy relative to the total time spent on events, targets, outcomes, and strategies, respectively (e.g., hours spent on the event of ‘preparing training materials’ / total hours spent on all events). We focus on the top five most time-consuming events, targets, outcomes, and strategies.

For Aim 2b, the amount of time spent on each event, target, and implementation strategy was calculated for each of the three most time-intensive intended outcomes: adoption, penetration, and fidelity. Then, we identified the events, targets, and strategies that took 10% or more of facilitators’ time for each of these outcomes. Next, the proportion of time spent on each event, target, and strategy for a given outcome was calculated by taking the time spent on each event, target, or strategy divided by the total time allocated to all events, targets, or strategies, respectively, for a given outcome. For instance, to calculate the proportion of time devoted to a specific event directed toward adoption, the formula was: hours spent on specific event directed toward adoption / total hours spent on all events directed toward adoption.

[1] The original variable options can be found on the ‘Lists + Definitions’ tab of the example log in the Supplement. These original options included ‘recording’ instead of ‘Zoom.’ However, facilitators alerted the research team that Zoom was a more accurate descriptor. Thus, for analysis, ‘recording’ was recoded as ‘Zoom.’

## Results

### Aim 1: Facilitator Perceptions of the Implementation Log

#### Deductive Coding

##### Acceptability, Appropriateness, Feasibility.

Almost all facilitators thought the log was appropriate (*n* = 5 out of 6 total facilitators). However, most facilitators did not find the log to be acceptable (*n* = 4) or feasible (*n* = 5).

#### Inductive Coding

See Table 1 for categories, themes, counts, and representative quotes.

##### Positive Qualities/Likes.

Most facilitators felt the log was valuable and would contribute meaningful knowledge to the field (*n* = 4). Many of the facilitators also liked the templates provided to help them complete the log (*n* = 4). Half said they liked the standardized variable options (*n* = 3) and observed that the log became faster and easier over time (*n* = 3). One facilitator appreciated that they were able to ask questions to get support with the log as needed (*n* = 1). One facilitator said they liked the log and had no suggestions for improvement (*n* = 1).

##### Barriers/Dislikes.

Almost all facilitators said that the log, as designed, did not fit with the flow of facilitation work (*n* = 5). Most facilitators said that a major barrier to completing the log was conflicting work priorities (*n* = 5), many of which felt more urgent than the Implementation Log.

With respect to the log itself, the most common critiques were that it was time consuming (*n* = 5) and did not always capture the most important facilitation or implementation activities (*n* = 5). Some facilitators felt the log was tedious (*n* = 2) and anxiety-provoking and overwhelming (*n* = 2). Additionally, facilitators had difficulty remembering or choosing between all the options presented (*n* = 3). They also struggled to learn how to use the log (*n* = 2) and with juggling their preferred approaches to organization (e.g., calendars, notes) versusadding to the log (*n* = 2). Most facilitators critiqued the format (*n* = 4) and half noted concerns about reliability and potential subjectivity (*n* = 3).

##### Suggestions for Improvement.

Most facilitators said that it would have been helpful if the log had used an automated tracking system (*n* = 5) and had been more streamlined, faster, and less complicated (*n* = 4). Facilitators offered specific suggestions, such as shifting the log to a survey or shared document (*n* = 3) or designing a system that merged with their calendars (*n* = 2). Additionally, facilitators suggested modifying the log to better capture the big picture (*n* = 1), community partners’ responses and outcomes (*n* = 1), and categorization of events (*n* = 4), including more granularity (*n* = 2). Two facilitators said that it would have been helpful to track by CMHC’s counties and then fill in the relevant variables (*n* = 2). Half of the facilitators requested standardized entries that filled in the relevant variables for repeated activities (e.g., recurring meetings) (*n* = 3). Facilitators also suggested that it would be helpful if they could write up a narrative of what they had done each day (*n* = 1) or use their own language to describe each activity (*n* = 2).

Facilitators suggested offering additional training (*n* = 3), a brief guide (*n* = 1), ongoing supervision or consultation (*n* = 1), and more information about how the data would be used so they knew which aspects of the log were most important (*n* = 1). They also said that protected time to complete the logs would have been helpful (*n* = 3).

### Quantitative Demonstration

#### Aim 2a: Time associated with events, targets, outcomes, and implementation strategies

A total of 3,752.7 hours were tracked for events. The top five most time-consuming events, targets, outcomes, and strategies are reported below in descending order (with separate analyses conducted for Aim 2b). See Table 2 for the specific number of hours and relative proportions of time.

For *events*, the most time was spent on internal team meetings for ongoing problem solving and sharing updates; communicating with CMHC providers about research-related logistics and study administration (e.g., helping providers complete surveys); preparing training materials; delivering trainings and workshops; and assessment supervision for study assessors. For *targets*, the most time was spent on executing; reflecting and evaluating; knowledge and beliefs about the intervention; engaging; and other personal attributes. For *intended outcomes*, the most time was spent on adoption; penetration; fidelity; feasibility; and appropriateness. For *implementation strategies*, the most time was spent on facilitation; problem identification; simplifying and clarifying communication wherever possible; providing ongoing consultation; and assessing for readiness and identifying barriers and facilitators.

#### Aim 2b: Time spent on events, implementation strategies, and targets for the three most time-intensiveintended outcomes

Below, the events, targets, and strategies that took 10% of time or more for each of the three most-time intensive outcomes (adoption, penetration, and fidelity) are reported in descending order. See Tables 3, 4, and 5 for the number of hours and relative proportions of time.

For *adoption*, facilitators logged 1,060.8 hours. Of the time directed toward adoption events, the events that took 10% or more of time were: delivering training and workshops; preparing training materials; communicating with CMHC providers about patient recruitment; and communicating with CMHC leadership about patient recruitment. The targets that took 10% or more of time spent on adoption targets were: executing; knowledge and beliefs about the intervention; and engaging. The implementation strategies that took 10% or more of time spent on adoption strategies were: facilitation; simplify/clarify communication wherever possible; develop educational materials; and make training dynamic (see Table 3).

For *penetration*, facilitators logged 785.5 hours. Of the time directed toward penetration events, the events that took 10% or more of time were: internal team meetings for ongoing problem solving and sharing updates, and strategizing and planning for ongoing county problem solving (the lead facilitator’s unique event). The target that took 10% or more of time spent on penetration targets was: reflecting and evaluating. The implementation strategies that took 10% or more of time spent on penetration strategies were: problem identification; goal setting; and assess for readiness and identify barriers and facilitators (see Table 4).

For *fidelity*, facilitators logged 507.5 hours. Of the time directed toward fidelity events, the events that took 10% or more of time were: assessment supervision and provider supervision. The targets that took 10% or more of time spent on fidelity targets were: executing, and knowledge and beliefs about the intervention. The implementation strategies that took 10% or more of time spent on fidelity strategies were: provide clinical supervision, provide ongoing consultation, simplify/clarify communication wherever possible, and audit and provide feedback. (see Table 5).

## Discussion

The goal of this report was to describe an Implementation Log developed in the context of a parent effectiveness-implementation trial to quantitively track a range of key implementation variables in near real time. Additionally, we assessed facilitator perceptions of the log and demonstrated ways the log data could be used to quantify implementation variables. The Implementation Log herein complements existing tracking approaches that focus on detailing a facet of implementation (e.g., adaptations), utilize archival data, or leverage qualitative coding (e.g., qualitative coding of the log or meeting notes) for analysis. Indeed, this log could be used independently to gather quantitative data, or paired with an existing tracking approach (e.g., tracking adaptations through FRAME-IS or the Dynamic Adaptations Process (20,23)) to yielda detailed, mixed methods picture of implementation processes in a given project. Importantly, variables selected for the log were derived from feedback and piloting with study personnel (i.e., facilitators) and existing frameworks, taxonomies, and guidelines (4,33,34,36). This multi-pronged theory-based approach was to help ensure that the log was informed by participatory principles (31) *and* grounded in established implementation science concepts and terminology to enhance consistency within implementation reporting.

### Qualitative Findings

In the qualitative feedback, facilitators appreciated the value of the log to contribute important knowledge to the field. However, there were three main suggestions for improvement: (1) automatize the log’s format, (2) offer additional training and supervision, and (3) improve flexibility to adjust the variable options.

First, most facilitators would have preferred a more automatized format to reduce the burden of tracking and help integrate tracking into facilitation workflows. This is consistent with Walsh-Bailey et al. (26), who found that end-users appreciated a structured, pre-populated format for implementation tracking. Although a structured, pre-populated format was used in our Implementation Log, the facilitators offered innovative ideas to enhance automaticity, such as utilizing a platform that could merge with work calendars or sending a daily survey with prepopulated entries for repeated activities. Second, like prior findings (26), several facilitators said that more initial training and ongoing supervision would have helped address challenges, questions, and uncertainties (13). Third, a piece of feedback that fell under both facilitator likes *and* dislikes was the variable options. Half of facilitators liked the standardized variable options offered by the log. However, more than half said the variables did not capture the most important aspects of facilitation or implementation. Facilitators suggested modifications, such as adding variables to better capture the big picture, community partners’ responses, as well as more granularity. As a next step for the log, future research could explore methods that support facilitators in adding their own variable options over time while maintaining rigor and standardization – for instance, discussing new variable options in supervision and, with group consensus, adding them to the log. Together, these suggestions of automatizing the format, adding more training and ongoing supervision, and allowing more flexibility to modify the log’s variables may improve end-user perceptions of the Implementation Log while preserving aspects that the facilitators appreciated (e.g., value to the field).

### Demonstration: Quantitative Results

There are many ways that the log data – including future iterations that incorporate the facilitator feedback – could be analyzed. In our demonstration, we first quantified the most time-consuming events, targets, outcomes, and implementation strategies. Critically, laying out the time needed for these different dimensions can help other teams develop detailed and empirically-informed implementation blueprints (34). This is key to ensuring that implementation leads – whether the PI of a research trial or a manager in a healthcare system – design teams, assign tasks, and allocate resources to most efficiently execute an implementation project.

Second, we identified the events, strategies, and targets for the most time-intensive outcomes – specifically, adoption, penetration, and fidelity. Given the relatively little empirical data on relationships between implementation variables (6), this type of analysis may help the field derive quantitively-grounded hypotheses. For instance, looking at penetration, facilitators allocated the most time to internal team meetings (event), reflecting and evaluating (target), and problem identification (strategy). Thus, a next step could be empirically testing whether these time-intensive events, targets, and strategies do indeed predict the outcome of penetration. In turn, such results may help other implementation teams identify which events, targets, and strategies to prioritize to maximize outcomes and/or help teams identify ways to strengthen the outcomes of a given project (e.g., if resources were poured into an event that did not actually predict the desired outcome). Together, the range of possible analyses afforded by the Implementation Log can help advance research on – and ultimately, understanding of – complex implementation processes.

There were important limitations that should be considered. First, the quantitative results do not include the first 10 months of the implementation phase, which were tracked in the pilot log. We encourage readers to interpret the quantitative results as a demonstration of analyses that *could* be conducted using the log, rather than generalizable results. Second, as described in the Method, to minimize burden for facilitators, we merged data from two other trackers maintained by facilitators (8.1% of the total data). This could be addressed in future research by using a single log that tracks all variables. Third, the sample size for our interviews was limited by the number of facilitators in the parent trial (*N* = 6). It is possible that more themes would have emerged with additional facilitators. Fourth, we sought to offer a ‘ready-made’ tool for tracking a range of implementation variables. However, other teams using the log may need to tailor the ‘events’ column prior to use, such that the list reflects the most common activities for the relevant implementation personnel.

## Conclusions

We present an Implementation Log used to track a range of key implementation variables in near real time. Per qualitative interviews, improvements could include automatizing the format, adding more training and ongoing supervision, and allowing study personnel flexibility to modify variable options. These recommendations could be incorporated while preserving the log’s primary contributions – namely, enabling teams to systematically report, analyze, and better understand the complexities of implementation.

## Supplementary Files

This is a list of supplementary files associated with this preprint. Click to download.

- AppendixA.docx
- ImpScienceImptLogPaperExampleLogSub2.xlsx
- ImpScienceImptLogPaperSupplementSub2.docx
- ImplementationLogChecklistSQUIRE2.0.docx

## Figures and Tables

**Table 1.** Categories, Themes, Coding Counts, and Representative Quotes from Inductive Coding of Facilitators Interviews

	**Count (N)**	**Representative Quote**
**Category: Positive Qualities/Likes**		
Valuable for the field	4	“I like that we’re doing something that helps people [to] have a blueprint in the future for what we did.” “I like that we are generating knowledge about how we did this process for people in the future.”
Liked templates	4	“I think that the decision [template] tabs were the most helpful for me.”
Liked variable options	3	“I like that we have a lot of variety of options for the tasks that we get to choose from. I think [LDS, 1^st^ author] did a really good job of identifying what are all of the different kinds of implementation tasks that you might do.”
Log became faster and easier over time	3	“I think once I found a system that worked well for me [when completing the log], I didn’t mind it so much.”
Able to ask questions to get support with log	1	“All of us would send [LDS, 1^st^ author] questions about the log whenever they came up, and then she would […] write back with her answers. And then she […] kept all of those questions and answers and uploaded them onto a [shared] document.”
Liked the log - no suggestions for improvement	1	“I think that the log was almost pretty perfect…I’m not sure if I have any suggestions. I liked the way that it was set up.”
**Category: Barriers/Dislikes**		
Did not fit with the flow of facilitation work	5	“I struggle[d] with the rapid switch tasking between lots of different things and the logs in order to keep up with them. In my experience [the log] requires that you stop what you’re doing and log whatever it is you’re doing throughout the day, right then.”
Conflicting work priorities	5	“My impression was that [the log] can get in the way of doing tasks especially when you are doing a lot of provider and client facing tasks…you’re like, okay, I’ll log this now, but you get another email that feels urgent so you do that instead.”
Time consuming	5	“It’s a pretty involved process that can get complicated […] I think the biggest barrier is that it does take a good amount of time sitting down to do it.”
Did not capture most important facilitation activities	5	“A lot of the things felt like this should be a something I log in the implementation log. But then it wouldn’t map on exactly to anything, or it didn’t feel like it was really serving a purpose for the community – that it was like a research-y thing. “
Tedious	2	“I think that processing just felt tiring at the end of the day. And so I remember feeling like I wanted to avoid it, to be honest.”
Anxiety-provoking and overwhelming	2	“I can imagine how important this data is […] So, I think I spent too much time because I want to make sure I’m capturing this as well as I possibly can.”
Difficulty remembering or choosing between all the options presented	3	“The definitions of them are sometimes a little bit hard to decipher. [… What if] I did part of that. Does that fully count?”
Struggled to learn how to use the log	2	“It took me a long time to get practice with using it. […] especially at the beginning, it took me so long, because I would have to look at the [tabs to] just making sure I am picking the right usages and scenario for things.”
Struggled with juggling their preferred approaches to organization (e.g., calendars, notes)	2	“The most challenging thing was just keeping track of all of the events in 2 different places, one in my calendar to make sure I have the time and then transferring it over.”
Critiqued the spreadsheet format	4	“I struggle with it being an Excel spreadsheet.”
Concerns about reliability and potential subjectivity	3	“We all [had] like, not drastically different, but slightly different ideas of what to put.”
**Category: Suggestions for improvement**		
Automated tracking system	5	“I’ve thought about automating the tracking […] So much of what we do is recorded over email or phone, and an app could [report, for example,] you sent 6 emails to this county. Then I could look through and put them in buckets or something.”
More streamlined, faster, and less complicated	4	“I think, the more streamlined and like, uncomplicated we can make the process, the more likely people are to be able to have space and time to carry them out.”
Shifting the log to a survey or a shared document	3	“I wish that they might be almost more in like a survey format. Something that I’ve thought is that it might be useful to transmit them to Qualtrics.”
Shifting the log to a system that merged with their calendars	2	“I would envision something more like a calendar format almost where you’d be able to add in an event. […] Like Google Calendars pops up event, location, time, place, and you have drop down [menu]. And if those strategies that you had already bundled together would auto populate, based on the event that you selected. And then there was like the option to edit, if needed.”
Modifying the log to better capture the big picture	1	“[Logistical tasks] are important for keeping things moving, but just from a facilitation standpoint I feel like right now the log is kind of weighted almost more towards logistics and less towards big picture.”
Modifying the log to better capture the community partners’ responses and outcomes	1	“I think it would be interesting to capture the responses that the community had toward […] specific effort or event. Were they receptive? Was it successful?”
Modifying the log to better capture activities	4	“It’d be nicer if there were more events […] that I actually do.”
Modifying the log to include more granularity within categories	2	“There’s things that I wish […] even more granular, for instances the provider incentives in general. It might be interesting to know: is certification more important than giftcards?
Track by county and then fill in the relevant variables	2	“One idea could be if I had like a list of the counties and then, like underneath them [we have] the different kind of big facilitation activities, and then putting down a time estimate spent on each one. So it was for example, ‘in meeting to discuss barriers and strategies around Monterey for 20 min’, or ‘emails with leadership problem solving trainings’ [and then] finding or assigning buckets per county, would feel easier.”
Standardized entries for repeated activities	3	“So having a central system [and] these are the basics. […] For example, every single Monday, we’re going to have this meeting, and then just assume it’s going to happen. And then if it doesn’t happen, we cut that meeting. So one less thing to think about or add to the log, which may not seem like a lot, but […] over time, it does add up.”
Narrative of what they had done in a given day	1	“I might be inclined to try to think about something that was more big picture, and maybe link [activities] to a story, writing a narrative, like a therapy note.”
Use their own language to describe each activity	2	“I would prefer to like write in your own activities in whatever kind of like language I wanted to, that would feel easier.”
Additional training at the start	3	“It could have been something as easy as like a little blurb like, ‘here’s the training document. This is what the implementation log is, this is why we’re doing it and this is how you can fill it out the way that we need it to be done. And also […] what are the ones that are really crucially important and some that could have some variability.’ “
A brief guide	1	“Having even just a like a quick short guide that will help inform [… and] give some insights on what the tabs are. Because, not anymore as much but beforehand, a lot of times I’d have to like reread every single template example tab and re familiarize myself just to make sure that I’m getting the most information which would add a lot of time.”
Ongoing supervision or consultation	1	“Having a scheduled time allows me to feel like I have the permission with myself to focus on it for like 20 minutes […] before the meeting […] to review prepare, and I think it will make me more actively thinking about the implementation and it might make it like the logs feel a lot easier to do. And then also just like answering questions […and] help with any potential differences that happen for a situation across facilitators.”
More information about how the data would ultimately be used	1	“For the facilitation data, […] I’m curious to know: what kind of analysis are going into it? And what kind of measures are going to be taken? Because there are some times when I’m filling out the log, where it’ll be almost identical in a row, except maybe one of the strategies is slightly different, just because it’s a slightly different topic. And so, I’m always curious how that’s differentiated and […] how unique they are?”
Protected time to complete the logs	3	“I there is a way to protect people’s time to do that in a routine fashion every day, like if there’s like 8:30AM every day, or whenever the work starts, and […] everybody just does it together for the day prior.”

*Note*. Count is the number of facilitators who endorsed each theme. […] indicates that select, original words from quotes were not included for readability or concision. [text] indicates words that were added or slightly rephrased by the 1^st^ and 2^nd^ authors to enhance readability and comprehension.

**Table 2.** Aim 2a: Time Spent on the Five Most Time-Intensive Events, Targets, Intended Outcomes, and Implementation Strategies

	Hours	Proportion of Event, Target, Outcome, or Strategy Time
**Events**	**3,752.7**	**100% of total event hrs**
Internal team meetings for ongoing problem solving and sharing updates	579.8	15.5%
Communicating with CMHC providers about research-related logistics and study administration	391.5	10.4%
Preparing training materials	361.4	9.6%
Delivering trainings and workshops	343.5	9.2%
Assessment supervision	289.3	7.7%
		
**Targets**	**3,752.7**	**100% of total target hrs**
Executing	1,756.9	46.8%
Reflecting and evaluating	705.7	18.8%
Knowledge and beliefs about the intervention	596.8	15.9%
Engaging	332.8	8.9%
Other personal attributes	133.6	3.6%
		
**Intended Outcomes**	**3,747.7**	**100% of total outcome hrs**
Adoption	1,060.8	28.3%
Penetration	785.5	21.0%
Fidelity	507.5	13.5%
Feasibility	364.7	9.7%
Appropriateness	197.8	5.3%
		
**Implementation Strategies**	**12,795.2**	**100% of total strategies hrs**
Facilitation	1,878.1	14.7%
Problem identification	1,511.7	11.8%
Simplifying and clarifying communication wherever possible	1,083.9	8.4%
Providing ongoing consultation	771.8	6.0%
Assessing for readiness and identify barriers and facilitators	756.4	5.9%

*Note*. Proportion of event, target, outcome, or strategy time is calculated as the time spent on each specific event, target, outcome, and strategy relative to the total time for the relevant category (e.g., time spent on a specific event / total number of hours spent on all events). A total of 3,752.7 hours were tracked for events and corresponding targets. A total of 3,747.6 hours were tracked for intended outcomes. A total of 12,795.2 hours were tracked for implementation strategies. Note that this number was much larger, because facilitators could select up to 4 strategies per event. Thus, to be conservative, we applied the amount of time reported for all strategies associated with a given event.

**Table 3.** Aim 2b: Time Spent on the Most Time-Intensive Events, Targets, and Implementation Strategies for Adoption

	Hours	Proportion of Event, Target, or Strategy Time
**Adoption**	**1,060.8**	**100% of total adoption hrs**
***Events***	***1,060.8***	***100% of adoption event hrs***
Delivering trainings and workshops	343.5	32.4%
Preparing training materials	331.4	31.2%
Communicating with CMHC providers about patient recruitment	154.8	14.6%
Communicating with CMHC leadership about patient recruitment	124.7	11.8%
***Targets***	***1,060.8***	***100% of adoption target hrs***
Executing	588.8	55.4%
Knowledge and beliefs about the intervention	316.6	29.8%
Engaging	138.7	13.1%
		
***Implementation Strategies***	***3,780.7***	***100% of adoption strategy hrs***
Facilitation	866.0	22.9%
Simplifying and clarifying communication wherever possible	694.6	18.4%
Developing educational materials	661.4	17.5%
Making training dynamic	657.1	17.4%

*Note*. The proportion of time was calculated by dividing the time spent on each specific event, target, or strategy directed toward adoption divided by the total time allocated to all events, targets, or strategies, respectively, for adoption.

**Table 4.** Aim 2b: Time Spent on the Most Time-Intensive Events, Targets, and Implementation Strategies for Penetration

	Hours	Proportion of Event, Target, or Strategy Time
**Penetration**	**785.5**	**100% of total penetration hrs**
***Events***	***785.5***	***100% of penetration event hrs***
Internal team meetings for ongoing problem solving and sharing updates	467.5	59.5%
Strategizing and planning for ongoing county problem solving (lead facilitator’s unique event)	234.3	29.8%
		
***Targets***	***785.5***	***100% of penetration target hrs***
Reflecting and evaluating	701.7	89.3%
		
***Implementation Strategies***	***2,512.0***	***100% of penetration strategy hrs***
Problem identification	708.5	28.2%
Goal setting	479.2	19.1%
Assess for readiness and identify barriers	474.1	18.9%

Note. The proportion of time was calculated by dividing the time spent on each specific event, target, or strategy directed toward penetration divided by the total time allocated to all events, targets, or strategies, respectively, for penetration.

**Table 5.** Aim 2b: Time Spent on the Most Time-Intensive Events, Targets, and Implementation Strategies for Fidelity

	Hours	Proportion of Event, Target, or Strategy Time
**Fidelity**	**507.5**	**100% of total fidelity hrs**
***Events***	***507.5***	***100% of fidelity event hrs***
Assessment supervision	288.5	56.9%
Provider supervision	133.8	26.4%
		
***Targets***	***507.5***	***100% of fidelity target hrs***
Executing	370.1	72.9%
Knowledge and beliefs about the intervention	133.8	26.4%
		
***Implementation Strategies***	***1,813.0***	***100% of fidelity strategy hrs***
Provide clinical supervision	421.5	23.2%
Provide ongoing consultation	421.5	23.2%
Simplify/clarify communication wherever possible	337.8	18.6%
Audit and provide feedback	287.4	15.9%

*Note*. The proportion of time was calculated by dividing the time spent on each specific event, target, or strategy directed toward fidelity divided by the total time allocated to all events, targets, or strategies, respectively, for fidelity.

## Data Availability

The datasets used and analyzed during the current study are available from the corresponding author on reasonable request.

## Abbreviations

CMHCs: Community mental health centers
TranS-C: Transdiagnostic Intervention for Sleep and Circadian Dysfunction (TranS-C)
U.S.: United States
SQUIRE: Standards for QUality Improvement Reporting Excellence
ERA: Emma R. Agnew
MD: Marlen Diaz
KF: Krista Fisher
REH: Rafael Esteva Hache
JS: Julia Spencer
SAH: Shayna A. Howlett
PI: Principal Investigator
AGH: Allison G. Harvey
CFIR: Consolidated Framework for Implementation Framework
UC Berkeley: University of California, Berkeley
ERIC: Expert Recommendations for Implementing Change taxonomy
LDS: Laurel D. Sarfan
LG: Linyan Ge
HIPAA: Health Insurance Portability and Accountability Act
Hrs: Hours
